# The Genetic Profile of Combat Sport Athletes: A Systematic Review of Physiological, Psychological and Injury Risk Determinants

**DOI:** 10.3390/ijerph21081019

**Published:** 2024-08-02

**Authors:** Konstantinos Anastasiou, Mhairi Morris, Liz Akam, Sarabjit Mastana

**Affiliations:** School of Sport, Exercise and Health Sciences, Loughborough University, Loughborough LE11 3TU, UK; konstantinos.anastasiou7@gmail.com (K.A.); m.a.morris@lboro.ac.uk (M.M.); e.c.akam@lboro.ac.uk (L.A.)

**Keywords:** systematic review, combat sports, physiological and psychological factors, injury susceptibility, PRISMA

## Abstract

This systematic review aims to assess the genetic determinants influencing combat sports performance and address potential gaps in previous reviews. Twenty-four selected studies were analysed, investigating genetic influences on physiological performance, psychological traits, psychophysiological factors like pain perception, and injury susceptibility in combat sport athletes. The systematic literature search, using keywords, encompassed PubMed, Scopus, SportDiscus, Medline, and Google Scholar. The Covidence systematic review management software facilitated the screening process and the creation of the PRISMA flow diagram. The quality assessment complied with the PRISMA guidelines, featuring a custom 10-point scale and the STREGA criteria for more reliable study inclusion. Collectively, the 24 studies incorporated 18,989 participants, of which 3323 were combat athletes of majority European ancestry (71.7%) from various combat sports disciplines. Twenty-five unique genetic variants were significantly associated with combat sports performance across diverse domains. These included physiological performance (nine genetic variants), psychological traits (ten genetic variants), psychophysiological factors (one genetic variant), and injury susceptibility (four genetic variants). In conclusion, this systematic review lays the foundation for a more comprehensive exploration of the association between genetics and athletic performance in the demanding arena of combat sports, offering valuable insights for talent identification, training optimisation, and injury prevention.

## 1. Introduction

### 1.1. Combat Sports

**Figure 1:**
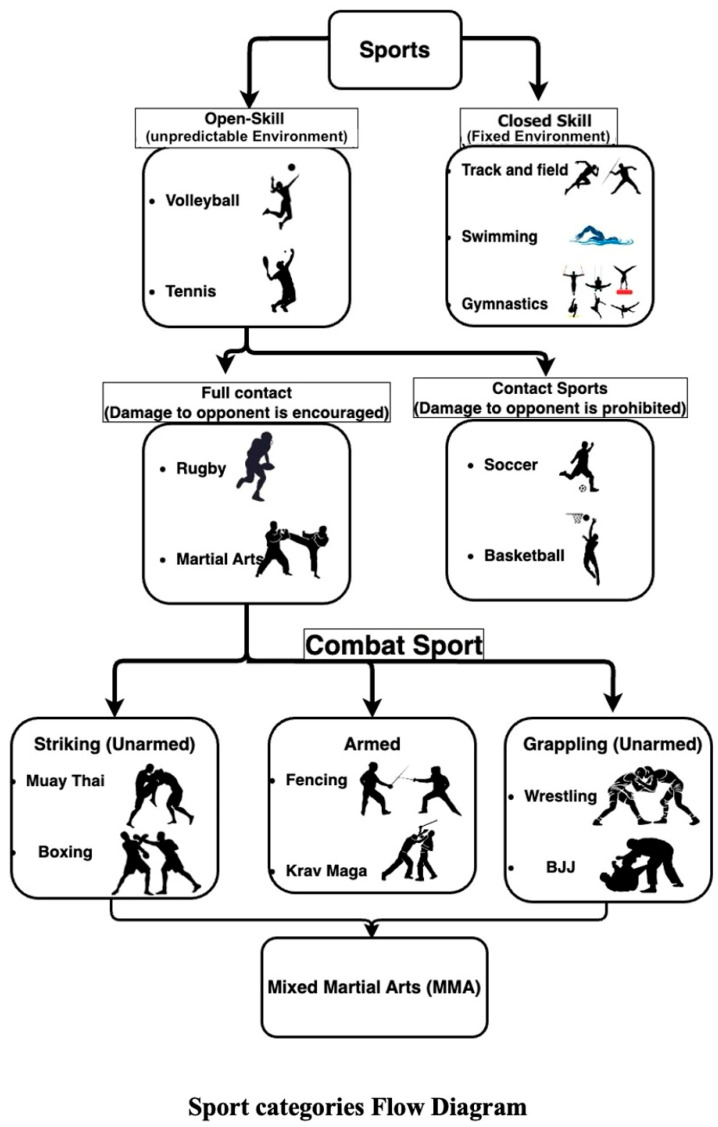
The chart demonstrates the different categories of open-skill sports and how sports are differentiated from non-contact to full contact and finally to full-contact mixed martial arts; BJJ: Brazilian jiu-jitsu.

Martial arts and combat sports boast a venerable history spanning millennia, dating back to the early development of weapons for hunting and defence approximately 30,000 years ago. The establishment of the Olympic Games in 776 B.C. laid the foundation for organised sports, including combat sports such as wrestling, boxing, and the precursor to today’s mixed martial arts (MMA), Pankration. Combat sports can be considered to encompass three main categories: weapons-based, grappling, and striking. Open-skill sports are commonly performed in a dynamic and changing environment, while closed-skill sports take place in a more predictable and static environment [1]; thus, combat sports are considered open-skill sports, dependent on a diverse range of attributes. Combat sports demand adaptability in unpredictable environments and quick decision-making in response to external stimuli [2,3]. Psychological stress has a more pronounced impact on combat sports than on closed-skill sports (CSS) [4]. Moreover, unlike controlled-contact sports like soccer, combat sports, described as collision sports, deliberately inflict damage on opponents for victory [5] (Figure 1). This tactical approach, leveraging injury as a tool, creates a higher-stress environment [6]. This distinct psychological component, coupled with the increased injury susceptibility, might be linked to specific genetic polymorphisms that can favour certain athletes by reducing the injury risk and increasing the mental toughness of high-performing fighters.

### 1.2. The Determinants of Combat Sports Performance

MMA’s complexity emerges from physiological, psychological, and anthropometric attributes. Physiologically, superior strength, neuromuscular power, and anaerobic and aerobic capabilities define successful athletes [7]. Grapplers emphasise longer-term anaerobic efforts, while striking specialists excel in shorter-term efforts. At the same time, both need a high aerobic capacity to sustain the high intensity and recover faster between rounds. This can be achieved by faster creatine phosphate resynthesis, which is directly linked to aerobic ATP synthesis [8]. Varied types of strength—isometric, explosive, and reactive—underpin combat techniques like submission, takedowns, and strikes, respectively [9]. Elite grapplers prioritise non-combat training, showcasing the primacy of physiological determinants over technical aspects [10]. Moreover, wrestlers’ muscular endurance is higher than that of their striking combat counterparts or jiu-jitsu practitioners, suggesting a higher physiological contribution in grappling sports [11].

Combat athletes manifest elevated self-esteem and reduced neuroticism, while team sports competitors display heightened conscientiousness [12]. Psychological resilience (mental toughness), linked with inherited traits, is important [13], especially in combat sports, where injury is an anticipated outcome [5]. The reaction time, an essential psychological attribute, significantly differs between elite and non-elite combat athletes by about 10%, while elite athletes are 50% less likely to make incorrect decisions [14]. Additionally, in a non-full-contact martial artist, hostility and general aggression are present at a statistically significantly lower level than in combat sports athletes [15,16,17].

Anthropometric and physical attributes like body composition, wingspan (i.e., the distance measured from the tip of one hand to the tip of the other hand when the arms are fully extended horizontally) [18], and flexibility are vital in combat sports [19,20]. High-level strikers’ augmented flexibility and mobility extend their tactical arsenal with high kicks and agility, helping with better positioning during grappling [9]. Intricacies such as wingspan and handedness hold dominance, with left-handedness conferring a notable advantage, underscoring the interplay between inherent traits and performance [18,21].

### 1.3. Nature vs. Nurture in Combat Sports Performance

The interplay of genetics and training in shaping athletic performance has long interested both athletes and academics. While physiological traits can be inherent, their activation often requires environmental stimuli. At the same time, personality traits can lean towards social influences. Both elements are changeable, but their functional effect is predetermined, with genetics significantly affecting the speed and potential peak level that can be reached [22]. Specific sporting practices can modulate gene expression through epigenetic alterations; for example, resistance training stimulates the expression of genes like Insulin-Like Growth Factor 1 (IGF-1) and myostatin, which regulate muscle growth and differentiation [23]. Most sporting practices offer the potential for gene modulation, with the repetitious and fixed nature of closed-skill sports commonly providing more pronounced gene expression alterations than in open-skill sports [22]. A meta-analysis reported that deliberate practice accounted for 26% of the variance in games, 21% in music, and 18% in sports performance [24]. Identical twin research indicated the substantial role of genetics, explaining ~80% of the variance in athletic performance [25] and up to 72% of the variance in VO_2_max [26].

Genetics and training contribute to an athlete’s performance; genetics predominantly shape the speed and potential magnitude of improvement. The impact of deliberate practice is notable, especially in predictable activities. Overall, the interaction of these factors intricately outlines the distinction between experts and novices in sports performance [22].

### 1.4. Genetic Polymorphisms and Sport Performance/Athletic Status

The completion of the Human Genome Project (HGP) in 2003 created a range of research opportunities for both medical and sports scientists [27,28]. Subsequent research endeavours have aimed at objectively outlining human groupings, particularly within the contexts of chronic diseases and elite athletic performance, with the identification of many disease- and sports performance-associated genes [27,28,29,30]. Initial efforts identified 187 genes linked to athletic prowess by 2005, later expanding to 239 genes [29]. However, the count decreased to 220 genetic polymorphisms by 2020 due to inconclusive case–control trials [27]. Presently, 252 DNA polymorphisms are associated with athlete status, with 128 markers displaying positive associations in at least two studies [28]. Notably, research has predominantly focused on polymorphisms related to physiological capacity, with limited investigations into psychological or injury-related variants. Recent genetic investigations have found 37 polymorphisms linked to sport-related injuries, encompassing muscle injuries (21), tendon and ligament injuries (7), and stress fractures (10) [30]. The identification of relevant polymorphisms could guide talent identification, aiding in the early recognition of promising athletes and even enabling the assessment of the genetic potential of professional fighters. Moreover, insights into the genes pivotal for combat success can shed light on which primary determinants (physiological, psychological, or others) prevail within this multifaceted domain.

### 1.5. Bridging the Gaps in Combat Sports Genetics

Given the complex and unpredictable nature of open-skill combat sports [22] (Georgiades et al., 2017), coupled with the heightened psychological stress and inherent injury susceptibility, a comprehensive investigation into the genetic determinants underlying these attributes is both warranted and essential. Previous systematic reviews had limited coverage of combat athletic determinants or only focused on specific sports and left gaps in the literature [31,32]. This systematic review aims to fill these gaps in the literature and provide a deeper understanding of the genetic influences that affect combat athletes’ performance and injury risk. The objectives include systematically reviewing and synthesising the existing literature on the genetic polymorphisms associated with various aspects of combat athlete performance and injury susceptibility across grappling, striking, and mixed martial arts disciplines.

## 2. Methods

### 2.1. Search Strategy

To comprehensively explore the relationship between genes and combat sports, a thorough search was conducted to address gaps not covered by previous reviews. The search scope encompassed studies from January 2011 to June 2023.

### 2.2. Databases and Keywords

The search strategy involved various databases, including PubMed, Scopus, SportDiscus, and Medline. Additionally, the first 20 pages of Google Scholar were extensively examined. The keywords employed in the search were methodically designed for broader bibliography identification: (((genes) OR (polymorphism)) OR (genome)) OR (“candidate genes”)) OR (genotype) AND (((((((((“martial arts”) OR (“combat sports”)) OR (“martial artists”)) OR (boxing)) OR (warrior)) OR (“mixed martial arts”)) OR (karate)) OR (taekwondo)) OR (kickboxing)) OR (wrestler)) OR (judo).

### 2.3. Study Selection Criteria

The study selection criteria were carefully designed following the PICOS framework [33]. Studies were considered for inclusion if they were published in English and employed case–control, cohort, or genome-wide association (GWA) designs. Selected studies focused on adults aged 18 and above, particularly healthy, high-level combat sports athletes such as those competing at the national, international, or Olympic levels. Comparator groups for analysis comprised lower-level combat athletes, non-contact sport athletes, and non-athletic populations.

The exclusion criteria comprised the following: studies not published in English, review articles, and cross-sectional studies lacking a control group. Furthermore, studies involving participants below 18 years of age, animal subjects, combat athletes below the national team level, or those with less than five years of experience compared to non-athletes were excluded. Also excluded were studies with no control groups and studies with combat participants numbering less than 20. Finally, studies with mixed groups, where the extraction of data specific to combat sports participants was not possible, were also excluded. It is noteworthy that the exclusion criteria mentioned above did not apply to injury-related studies due to the limited sources in the literature, necessitating a less selective approach.

### 2.4. Study Selection Process

**Figure 2:**
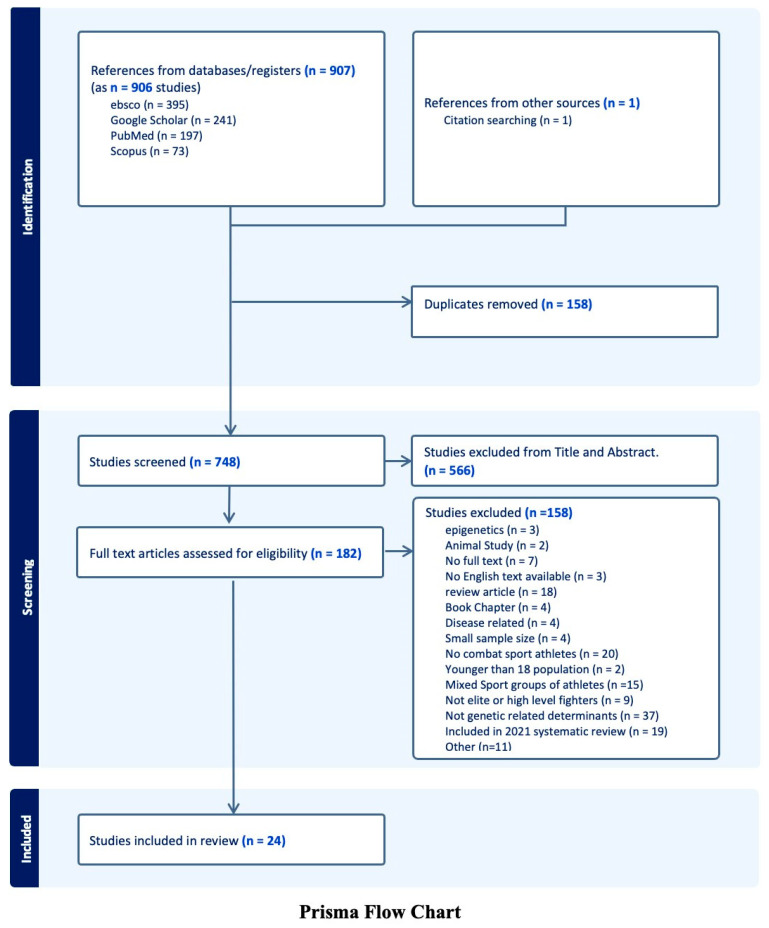
Data selection process according to inclusion and exclusion criteria and PRISMA guidelines. In total, 24 studies were selected for the review out of 907 articles. Duplicates were removed automatically, and the flow chart was created using the Covidence citation manager.

The Covidence Systematic Review software (Covidence Systematic Review software, 2023) [34] facilitated the screening process. Initial screening consisted of reviewing the titles and abstracts and a full-text examination by two reviewers (KA and SM). Any differences in evaluation between the two reviewers were resolved via the discussion of individual studies. After the careful elimination of duplicates, only full texts were considered for inclusion for data extraction. Finally, 24 studies were deemed suitable for this review, with the selection process displayed in Figure 2.

### 2.5. Data Extraction

Data extraction encompassed crucial elements of each included study, such as the first author, publication date, study design, participant characteristics (such as gender, sport, competition level, and ethnicity), the genetic polymorphisms analysed, and the reported statistical outcomes. An Excel spreadsheet was used for data extraction, and Table 1, Table 2 and Table 3 contain all of the details from the data extraction process.

### 2.6. Quality Assessment

Quality assessment was performed using a custom 10-point scale, aligning with the Strengthening the Reporting of Genetic Association Studies (STREGA) guidelines [35].

## 3. Results

A total of 24 studies met the inclusion criteria and were included in the final review (Figure 2). The selected studies covered various aspects of combat sports genetics: ten explored physiological performance, eight looked into psychological factors, two investigated psychophysiological aspects (such as pain perception), and four focused on injury-related genetics. Eight studies published after 2021 were included, and twelve studies not included in the review of Youn et al. (2021) [31] were deemed suitable for this review and were included (Table 1).

Quality assessment was performed using a custom 10-point scale, aligning with the Strengthening the Reporting of Genetic Association Studies (STREGA) guidelines [35]. Out of these, 14 studies were rated as high quality (Score 8–10), while 10 studies were categorised as medium quality (Score 5–7) (Table 2).

**Table 1:** Summary of included studies.

	Reference	Performance Type	Participants	Design	Genes (SNPs)	Results
Previous Systematic Review	Youn et al. (2021) [31]		Total 14,313 Combat Sports Athletes = 2786, Other Sports Athletes = 2558 Non-Athlete Controls 8969	18 full-text articles	71 genes (109 SNPs)	17 SNPs significant Athletic Performance = 8 (*PPARA* rs4253778, *ACTN3* rs1815739, *ACE* rs4646994, *CKM* rs8111989, *MCT1* rs1049434, *FTO* rs9939609, *GABPβ1* rs7181866, and rs8031031) Psychological Traits = 5 (*COMT* rs4680, *FEV* rs860573, *SLC6A2* rs2242446, *HTR1B* rs11568817, *ADRA2A* rs521674) Additional: Reaction Time Related = 4 (*KIF27* rs10125715, *APC* rs518013, *TMEM229A* rs7783359, *LRRN3* rs80054135)
2021–2023 Studies	Leznicka et al. (2023) [36]	Psychophysiological/Pain Perception	Combat Athletes = 214 (Boxing, Karate, MMA)	CC	*SCN9A* (rs6746030)	Higher pain tolerance for GA and AA genotypes.
	Akazawa et al. (2022) [37]	Physiological	Combat Athletes = 94 (Judo, Wrestling, Boxing, Karate, Taekwondo, Fencing)	CC	*ACTN3 (R577X)*	RR frequency is higher in martial artists. R allele frequency increases in accordance with athletic status.
	Fichna et al. (2021) [38]	Psychological	Combat Athletes = 305 (Not Specified)	CC	*SLC6A2* (rs1805065) *SYNE1* (rs2635438)	Positive association of minor allele T with success in combat sports.
	Michałowska-Sawczyn et al. (2021) [39]	Psychological	Combat Athletes = 258 (MMA, Boxing, Judo, Wrestling, Karate, Kickboxing)	CC	*DRD2* (rs1799732, rs107656, rs1800498, rs6276, rs1079597) *ANKK1* (rs180049)	Higher CC genotype and major C allele in combat athletes.
	Niewczas et al. (2021a) [40]/Humińska- Lisowska et al. (2022) [41] (Merged)	Psychological	Combat Athletes = 153 (MMA, Boxing, Judo, Wrestling, Jiu-Jitsu, Karate, Kickboxing)	CC	*BDNF* (rs10767664/rs2030323/rs6265)	Significant combined effect for martial arts/control and Conscientiousness and Extraversion scale for all *BDNF* genes.
	Niewczas et al. (2021b) [42]	Psychological/Personality	Combat Athletes = 85 (MMA)	CC	*DRD2* (rs1799732)	Significant effect of *DRD2 rs1799732* genotype on MMA participants’ control and reward dependence.
	Peplonska et al. (2021) [43]	Psychological	Combat Athletes = 308 (Not Specified)	CC	*MYRF (*20 SNPs) *SOX10* (7 SNPs) *OLIG2* (rs762178)	*MYRF* minor alleles rs7943728 and rs61747222 showed a correlation with higher levels of sports achievement.
	Ponzi et al. (2021) [44]	Psychological	Combat Athletes = 65 (Karate)	Cohort	*SLC6A4* (5HTTLPR)	Salivary cortisol levels 10 min before the competition were higher in losers and in athletes with the S allele.
Studies Before 2021	De Oliveira Rocha et al. (2020) [45]	Physiological	Combat Athletes = 28 (Wrestling, Taekwondo)	CC	*ACE I-D*	Higher I allele for male only fighters.
	Bondareva et al. (2019) [46]	Physiological	Combat Athletes = 36 (Sambo, Boxing, Judo, Taekwondo)	CC	*UCP1* (rs1800592) *UCP2* (rs660339) *UCP3* (rs1800849	Higher major allele *UCP1* (A) and *UCP2* (C) in martial artists compared to controls.
	Cherepkova et al. (2018) [47]	Psychological	Combat Athletes = 107 (MMA)	CC	*SLC6A4* (VNTR ID)	Higher D allele frequency in the groups of convicts and MMA fighters than controls.
	Leźnicka et al. (2018) [48]	Psychophysiological/Pain Perception	Combat Athletes = 99 (Boxing)	CC	*SCN9A* (rs6746030)	No difference in allele or genotype frequency.
	Batavani et al. (2017) [49]	Physiological	Combat Athletes = 172 (Karate)	CC	*CK-MM* (rs8111989)	Higher heterozygous AG genotype in professional than armature karateka athletes.
	Marziliano et al. (2017) [50]	Physiological	Combat Athletes = 117 (Muay Thai)	CC	*ACTN3* R577X (rs1815739)	Heterozygous RX genotype higher in Muay Thai fighters.
	Itaka et al. (2017) [51]	Physiological	Combat Athletes = 129 (Judo)	CC	*ACTN3* R577X (rs1815739) *ACE* I/D	*ACTN3 RR* genotype was correlated with faster wins in matches. No difference for the *ACE* gene.
	Bondareva et al. (2016) [52]	Physiological	Combat Athletes = 220 (Sambo)	CC	*EPAS1* (rs1867785)	Higher minor A allele in professional sambo wrestlers than controls.
	Itaka et al. (2016) [53]	Physiological	Combat Athletes = 129 (Judo)	CC	*ACE* I/D	No difference in frequency or athletes’ endurance.
	Butovskaya et al. (2015) [54]	Psychological	Combat Athletes = 106 (Wrestling, Judo, Sambo)	CC	*SLC6A4 5-*HTTL (rs25531) *MAOA* (VNTR) *5-HT1A* (rs6295) *5-HT2A* (rs6311)	A combined effect of the level of sports achievement and *5 HT2A* genotypes was found, as well as gender and *5 HT1A* genotypes, on the self-rating for conscientiousness.
	Hermine et al. (2015) [55]	Physiological	Combat Athletes = 34 (Wrestling)	CC	*HFE (*H63D, C282Y, S65C)	Very high *HFE* mutation frequency in international-level athletes and fighters compared to controls.
	Gabbasov et al. (2013) [56]	Physiological	Combat Athletes = 86 (Judo)	CC	*HIF1A* (rs11549465)	Higher 582Ser allele frequency in wrestlers and weightlifters than non-strength control subjects.
Injury-Related	Jowko et al. (2023) [57]	Bone Strength	Combat Athletes = 37 (Wrestling, MMA)	CC	*VDR* *ApaI,* rs 7975232 *BsmI,* rs1544410 *FokI* rs2228570 *COLIA1* rs1800012 *CALCR* rs1801197 *SOD1* rs2234694 *SOD2* rs4880 *GPx* rs1050450	*FokI* AG and *CALCR* AA genotypes associated with greater bone density response to sports training.
	Kwasniak et al. (2018) [58]	Joint or Spinal Injury	Combat Athletes = 108 (Judo)	CS	*ACE* (I/D) *ADRB2* (Arg16Gly, Gln27Glu) *MSTN* (K153R)	*ACE* I allele may predispose individuals to joint injuries and *ADRB2* Gln allele may protect individuals from spinal injuries.
	Banks et al. (2017) [59]	Concussion	Combat Athletes = 193 (Boxing, MMA, Other)	CC	*APOE*	No relationship was found.
	Koyama et al. (2017) [60]	Disc Degeneration	Combat Athletes = 215 (Wrestling)	CS	*COL11A1* rs1676486	*COL11A1* CC genotype was significantly correlated with cervical disc degeneration.

Note. MMA indicates mixed martial arts; CC indicates case–control; CS indicates cross-sectional; GWA indicates genome-wide association study; SNP indicates single-nucleotide polymorphism. Studies before 2021 indicate studies not found or not included in the review of Youn et al. [31].

**Table 2:** Quality assessment of included studies.

Lead Author	Date	Design	Case Selection (Max Score 3)	Case Sample Size (Max Score 1)	Control Selection (Max Score 1)	Control Sample Size (Max Score 1)	Gold-Standard Procedures (Max Score 1)	Sample Racial Homogeneity (Max Score 1)	HWE (Max Score 2)	Total Score (*/10)
Leznicka [36]	2023	CC	*	*	*	*	*	*	**	8/10
Akazawa [37]	2022	CC	**	*	*	*	*	*	*	8/10
Fincha [38]	2021	CC	***	*	*	*	*	*	**	10/10
Michałowska-Sawczyn [39]	2021	CC	**	*	*	*	*	*	_	7/10
Niewczas/Humińska-Lisowska (Merged) [40,41]	2022	CC	**	*	*	*	*	*	**	9/10
Niewczas [42]	2021	CC	**	*	*	*	*	*	**	9/10
Peplonska [43]	2021	CC	**	*	*	*	*	*	*	8/10
Ponzi [44]	2021	Cohort	*	*	*	_	*	*	_	5/10
De Oliveira Rocha [45]	2020	CC	**	_	*	_	*	*	*	6/10
Bondareva [46]	2019	CC	**	_	*	*	*	*	*	7/10
Cherepkova [47]	2018	CC	**	*	*	*	*	*	*	8/10
Leznicka [48]	2018	CC	***	*	_	*	*	*	**	9/10
Batavani [49]	2017	CC	**	*	*	*	*	*	_	7/10
Marziliano [50]	2017	CC	**	*	*	*	*	*	**	9/10
Itaka [51]	2017	CC	**	_	*	*	*	*	_	6/10
Bondareva [52]	2016	CC	***	*	*	*	*	*	_	8/10
Itaka [53]	2016	CC	**	*	*	*	*	*	**	9/10
Butovskaya [54]	2015	CC	***	*	*	*	*	*	_	8/10
Hermine [55]	2015	CC	***	_	*	*	*	*	_	7/10
Gabbasov [56]	2013	CC	***	*	*	*	*	_	**	9/10
Jowko [57]	2023	CC	*	_	*	*	*	*	_	5/10
Kwasniak [58]	2018	CS	**	_	*	*	*	*	*	7/10
Banks [59]	2017	CC	**	*	*	*	*	*	_	7/10
Koyama [60]	2017	CS	*	*	*	*	*	*	**	8/10

Note. A 10-scale customised quality assessment was created based on the current study’s inclusion criteria. Quality: High 8–10, Medium 5–7, Low 1–4. CC indicates Case–Control; CS indicates cross-sectional; Selection based on inclusion criteria. Ratings: 1 star for national-level athletes; 2 stars for professionals and international athletes; 1 additional star for higher-performing elite athletes. Adequate combat athletes sample size >50 and control >50. Racial homogeneity as reported by the “Strengthening the Reporting of Genetic Association Studies” (STREGA) [35]. HWE indicates Hardy–Weinberg Equilibrium. If in accordance with HWE, 1 star is allocated for controls and 1 for cases. A maximum of 2 stars can be awarded.

Overall, these 24 studies examined 31 genes and 70 polymorphisms, revealing a rich landscape of genetic variation within combat sports athletes. In conjunction with the findings from Youn et al.’s [31] review, which identified 109 SNPs in 55 genes, a comprehensive genetic profile for combat sports athletes has emerged. Together, the two reviews have identified a total of 77 unique genes and 176 SNPs in combat sport attributes. *ACTN3 R577X* (nine studies) and *ACE ID* (seven studies) were the two most commonly studied genes in combat sport genetic studies.

**Figure 3:**
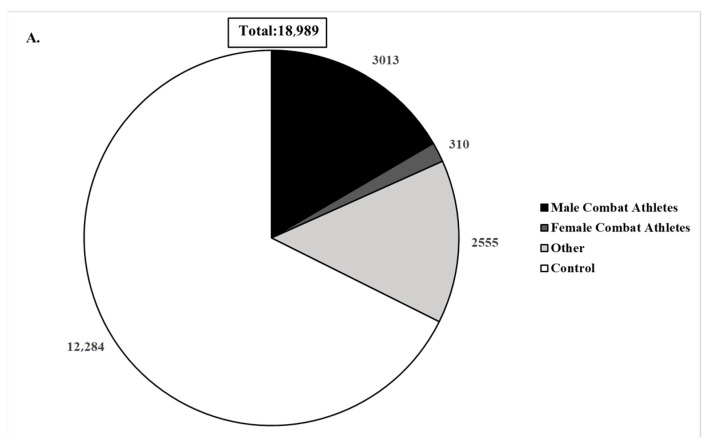

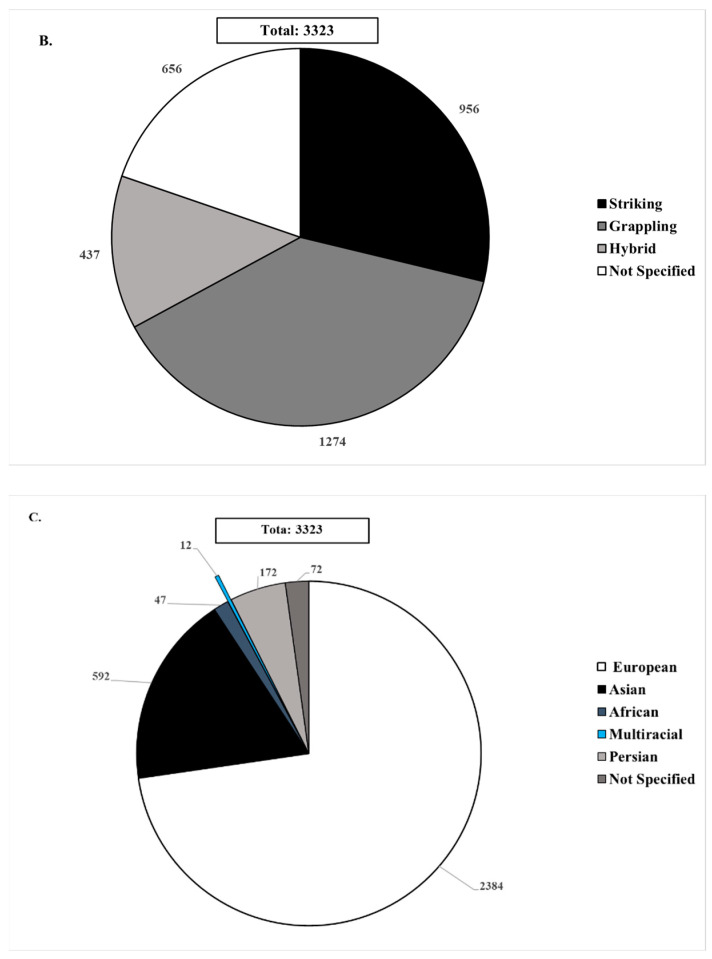
Pie charts demonstrating gender and sports distribution for all participants (**A**), combat sport type (**B**), and combat athletes’ ethnicities (**C**). Other indicates other non-combat sports (soccer, rugby, track and field, etc.); control indicates non-athletes; striking includes boxing, kickboxing, karate, taekwondo, and Muay Thai; grappling includes wrestling, sambo, judo, and jiu-jitsu; hybrid indicates mixed martial artists.

The participant cohort for the current review comprised a diverse population, with a total of 18,989 individuals. Among them, 3323 were combat sports athletes, with 956 engaged in striking sports (e.g., boxing, kickboxing, karate, taekwondo, Muay Thai), 1274 in grappling sports (e.g., wrestling, jiu-jitsu, sambo, judo), 437 in mixed martial arts (MMA), and 656 not specifying the discipline. Additionally, 2555 non-combat sports athletes and 12,284 non-athlete controls participated. The aforementioned findings and the combat athletes’ ethnic distribution can be seen in Figure 3.

Youn et al. [31] identified 13 SNPs significantly associated with combat sports performance and revealed four new SNPs through genome-wide association (GWA) studies, specifically related to wrestlers’ reaction times. In the current review, 24 variants (genotypes or alleles) demonstrated significant associations with physiological performance (nine SNPs), psychological factors (10 SNPs), psychophysiological traits like pain perception (one SNP), and injury susceptibility (four SNPs, with two being unique; Table 3). This cumulative evidence contributes to a growing catalogue of SNPs linked to combat sports participation, performance, and injury risk.

Seven SNPs were statistically associated with combat sports attributes in at least two studies (Table 3). These SNPs include *ACTN3* rs1815739 (power and strength R allele: 15 studies; endurance X allele: four studies); *ACE* (endurance I allele: 17 studies; power and strength D allele: 14 studies); *ADRB2* rs1042713 (endurance C allele: two studies; power G allele: two studies); *CK-MM* (endurance A allele: three studies; power and strength G allele: five studies); *UCP2* rs660339 (endurance T allele: three studies); *HFE* rs1799945 (endurance G allele: five studies); and *HIF1A* rs11549465 (endurance C allele: two studies; power and strength T allele: six studies). Four additional polymorphisms (*PPARA* rs4253778, *MCT1* rs1049434, *FTO* rs9939609, *GABPβ1* rs7181866) in Youn et al.’s [31] systematic review demonstrated statistical significance in at least two studies, bringing the total number of SNPs to 11.

**Table 3:** Comparison of allele and genotype frequency between combat athletes and controls.

Reference (Phenotype)	Candidate Allele or Genotype	Frequency in Combat Athletes	Frequency in Control	Statistics and *p*-Value
Jowko et al. (2023) [57] Injury ↓	*CALCR* AA genotype greater BMD response to sports training.	61.70%	50.60%	*p* < 0.05 *
Leznicka et al. (2023) [36] Psychophysiological	*SCN9A* rs6746030 GG genotype.	92.80%	66.70%	*p* = 0.02 *
	Higher pain tolerance than AG and AA.			
Akazawa et al. (2022) [37] Physiological	*ACTN3* rs1815739 R577X R/R genotype compared to control and endurance.	32%	20%	OR = 1.836, *p* < 0.05 *
Fichna et al. (2021) [38] Psychological	*SLC6A2* rs1805065 T allele modulating mood, arousal, memory, learning, and pain perception.	N/S	N/S	OR = 6.56, *p* = 0.010 **
Michałowska-Sawczyn et al. (2021) [39] Psychological/personality	*DRD2 rs1079597* C allele.	86.00%	82.00%	*p* = 0.034 *
Niewczas et al. (2021a) [40]/Humińska-Lisowska et al. (2022) [41] (Merged) Psychological	*BDNF* rs10767664 T/T genotype.	91.40%	81.60%	*p* = 0.044 *
	*BDNF* rs2030323 G/G genotype.	88.10%	69.30%	*p* = 0.029 *
Peplonska et al. (2021) [43] Psychological	*MYRF rs61747222* A allele less frequent than G allele.	N/S	N/S	OR = 0.6, *p* = 0.046 *
	*MYRF rs198459* A allele is less frequent than the G allele.	N/S	N/S	OR = 0.58, *p* = 0.046 *
Ponzi et al. (2021) [44] Psychological	*SLC6A4* 5HTTLPR S/S genotype has higher cortisol than the L/L genotype.	N/S	N/S	F = 4.51 *p* < 0.05 *
Bondareva et al. (2019) [46] Physiological	*UPC1* A allele higher than G allele.	83.30%	Other Sports = 70.1%	*p* < 0.05 *
	*UPC2* C allele higher than the T allele, reduced oxidative stress from aerobic metabolism. Thus, higher lactate tolerance.	76.40%	Control = 59.3%	*p* < 0.05 *
Cherepkova et al. (2018) [47] Psychological/aggression	*SLC6A4* (ID) D/D genotype,	20.80%	3.90%	OR = 6.5, *p* < 0.0001 ***
	*SLC6A4* VNTR 10/12 genotype.	55.7%.	45.50%	*p* < 0.003 **
Kwasniak et al. (2018) [58] Injury ↑	*ACE* I/D genotype higher performance.	Elite = 66.7	Non-elite = 39.5%	*p* < 0.05 *
	*ADRB2* Glu27Gln genotype higher performance.	Elite = 83.3%	Non-elite = 63.2%	*p* < 0.05 *
	*ACE I/I* genotype increased joint injuries.	Non-injury = 4.3%	Injury group = 33.8%	OR = 6.1, *p* = 0.005 **
	*ADRB2* Glu27Gln genotype increased spinal injury risk.	Non-injury group = 13.1%	Injury group = 25.0%	*p* = 0.03 *
Batavani et al. (2017) [49] Physiological	*CK-MM* A/G genotype better performance.	56.90%	43.00%	*p* < 0.05 *
Koyama et al. (2017) [60] Injury ↓	*COL11A1* (rs1676486) C/C genotype protective against disc degeneration.	No CDD group = 51.1%	CDD group = 37.8%	*p* = 0.035 *
Marziliano et al. (2017) [50] Physiological	*ACTN3* rs1815739, X allele higher.	32.00%	29.20%	*p* = 0.012 *
Itaka et al. (2017) [51] Physiological/power	*ACTN3* rs1815739, R allele faster in winning a match.	Fast winners = 82.0%	Slow winners = 16.7	*p* < 0.01 **
Bondareva et al. (2016) [52] Physiological/endurance	*EPAS1* (rs1867785) A allele.	Athletes = 38.2%	Non-athletes = 25.5%	*p* = 0.003 **
		Winners = 40.2%	Losers = 36.9%	*p* < 0.05 *
Butovskaya et al. (2015) [54] Psychology/personality	*5-HTTLPR* S/S genotype higher conscientiousness.	23.80%	11.00%	*p* = 0.04 *
Hermine et al. (2015) [55] Physiological	*HFE* mutations (H63D, C282Y, S65C).	N/S	N/S	OR = 3 *p* < 0.01 **
Gabbasov et al. (2013) [56] Physiological	*HIF1A* 582Ser allele.	15.70%	7.50%	*p* = 0.0002 ***

Note. Only studies with significant SNP frequencies are shown in the table (* *p*-value < 0.05); **↓** decreased injury risk; ↑ increased injury risk; OR indicates odds ratio; X^2^ indicates chi square; F indicates square value of mean difference; N/S indicates not specified. BMD indicates bone mineral density; CDD indicates cervical disc degeneration * Significance *p* < 0.05; ** Significance *p* < 0.01; *** Significance *p* < 0.001.

## 4. Discussion

### 4.1. Genetics in Combat Sports

This study aimed to explore the genetic impact on performance and injury risk in combat sport athletes through a systematic review. Twenty-four studies were scrutinised utilising a custom 10-point scale and adhering to the STREGA guidelines for quality assessment [35]. Of these, 14 received high scores (8–10), while 10 were rated as medium quality (5–7). These investigations delved into physiological, psychological, psychophysiological, and injury-related traits, scrutinising 31 genes and 70 polymorphisms. Youn et al. [31] found 109 SNPs in 55 genes; together, both reviews document 77 unique genes and 176 SNPs in combat sport attributes. Prominent genes investigated included *ACTN3 R577X*, *ACE ID*, and *COMT*. Semenova et al. [28] documented 251 polymorphisms related to athletic performance; however, their update did not specifically focus on combat sports. Semenova et al. [28] predominantly focused on power-, strength-, and endurance-related genetics. Given the diverse and multifaceted nature of combat sports, encompassing various disciplines with distinct demands and characteristics, the scope of performance determinants extends beyond those covered in their update.

Previous systematic reviews in the field had limitations in terms of depth and the coverage of determinants specific to combat athletes, leaving notable gaps in the literature [31]. Two additional systematic reviews also investigated the genetics of combat sports [32,61]. It is worth mentioning that both reviews encompassed studies that were also included in the Youn et al. [31] review, resulting in similar findings. Moreover, the most recent systematic review concentrated exclusively on taekwondo athletes [32] and incorporated studies where data extraction for taekwondo individuals was unavailable [45,62,63,64]. Furthermore, it is crucial to acknowledge that combat sports’ physiological and psychological determinants can significantly vary [65], emphasising the need for a tailored and sport-specific approach when studying the genetics of combat sports.

#### 4.1.1. Physiological Genetics

The present review identified certain SNPs associated with physiological mechanisms in combat sports, including *ACTN3* rs1815739, *ACE* I/D, *UCP1* rs1800592, *UCP2* rs660339, *CK-MM* rs1815739, *EPAS1* rs1867785, *HFE* rs1799945, and *HIF1A* rs11549465. The *ACTN3* gene, characterised by the R577X polymorphism, plays a pivotal role in fast-twitch muscle fibre development. This genetic variant, particularly the RR or RX genotypes, is closely associated with explosive movements and rapid muscle force generation, essential for power and strength athletes [66,67]. In parallel, the *ACE* gene’s I/D polymorphism influences the angiotensin-converting enzyme (ACE) levels in plasma. The I allele leads to decreased ACE levels, resulting in enhanced skeletal muscle vasodilation. This physiological adaptation facilitates an increased oxygenated blood supply to working muscles, a trait highly beneficial for endurance [68].

The significance of these genes aligns with the anaerobic nature of combat sports, where power and strength are critical. Studies have consistently reported elevated frequencies of the *ACTN3* R and *ACE* D alleles in power-focused athletes such as wrestlers [69,70]. Meta-analyses further underscore these associations [71,72], revealing a significantly lower *ACE* II genotype frequency (OR = 0.93 vs. 1.35 *p* < 0.01) and higher *ACTN3* RR genotype frequency (OR = 1.21 vs. 0.94 *p* < 0.01) between power and endurance athletes, respectively. However, it is worth noting that some studies have produced contradictory results [53,73], reinforcing the necessity for sport-specific meta-analyses.

Two promising genes, *CK-MM* and *EPAS1*, hold intriguing potential in combat sports genetics. The *CK-MM* gene encodes creatine kinase-M, a vital enzyme in ATP resynthesis during anaerobic activities [74]. A recent meta-analysis suggests an association between the *CK-MM* gene and anaerobic-dominant athletes, with a higher frequency of the G allele (OR, 1.14; *p* = 0.03) and GG genotype (OR, 1.54; *p* < 0.0001) compared to the control population [75]. This association indicates that specific genetic variations within the *CK-MM* gene could influence the muscle’s ability to perform under intense, short bursts of effort, a hallmark of many combat sports. On the other hand, *EPAS1* regulates genes involved in erythropoiesis and angiogenesis, crucial for delivering oxygen to muscles during endurance activities [76]. While not yet extensively explored in combat sports, the *EPAS1* gene and the remaining genes (*UCP2*, *HFE*, and *HIF1A*) hold promise.

#### 4.1.2. Psychological Genetics

This review highlights several SNPs related to psychological mechanisms, including *SCN9A* rs6746030, *SLC6A2* rs1805065, *DRD2* rs1079597, *BDNF* rs10767664 and rs2030323, *MYRF* rs61747222, rs198459, *5-HTTLPR* rs4795541, and *SLC6A4* VNTR. While these genes are implicated in psychological traits, it is notable that they had not been tested in meta-analyses for either combat sports or general athletic performance. These psychological-related genes can be grouped based on psychological determinants, including pain perception (*SCN9A*, *SLC6A2*), personality traits/mood (*SLC6A2*, *DRD2*, *SLC6A4, 5-HTTLPR*), and learning ability (*BDNF*, *MYRF*; Table 2 and Table 3).

One primary factor influencing learning appears to be *BDNF*, which controls neuroplasticity and the creation of new synapses [77]. It also influences personality traits, increasing conscientiousness and extraversion [40,41], while it may be linked to milder post-concussion symptoms [78]. However, *MYRF*, which plays a role in neuron myelination related to kinaesthetic learning [43], has not been extensively studied.

*SLC6A4, SLC6A2*, and *DRD2* are known to be involved in the serotonergic and dopaminergic systems [79], which impact the perception of exercise-induced fatigue [80] and anxiety [81]. During competition, individuals with the *SLC6A4* SS genotype showed higher pre-competition cortisol release and loss in karate competitions [44].

This systematic review identified one polymorphism in the *SCN9A* gene as an important factor in pain perception. SCN9A provides instructions for the alpha subunit of a sodium channel (NaV1.7) found in nociceptors (pain sensors). Mutations in this gene reduce pain sensitivity [82]. However, contrasting results exist, with the *SCN9A* GG genotype associated with an increased pain threshold and tolerance in one study [36] but not in a previous study [48]. Considering pain perception, it is crucial to note that while a reduced response to painful stimuli can provide advantages for combat athletes, it also poses a double-edged sword with an evolutionary purpose of warning and protection [36,83].

#### 4.1.3. Injury-Related Genetics

This review identified several SNPs related to injury, including CALCR rs1801197, ADRB2 rs1042714, and COL11A1 rs1676486. Variations in the CALCR gene may affect bone density and the ability of bones to repair themselves after an injury. This can influence the susceptibility to fractures and other bone-related injuries, as well as the healing process [84]. The ADRB2 gene, encoding the beta-2 adrenergic receptor (ADRB2), responds to stress hormones and enhances endurance performance by increasing fatty acid oxidation, which is the primary energy source during prolonged endurance exercise [85]. The mechanism by which ADRB2 contributes to the injury risk is unclear currently. Variants in the COL11A1 gene can affect the structural properties of cartilage, making individuals more prone to cartilage damage and joint injuries [86]. The emphasis of genetic studies in contact sports related to injuries should be on identifying a set of different SNPs that may help in reducing injuries and their impacts on athletes’ long-term careers.

### 4.2. Limitations, Future Directions, and Implications

The current genetic research in combat sports faces numerous limitations, underscoring the need for targeted future directions to enhance its applicability and reliability. Small sample sizes [44] and studies involving non-elite athletes [36,44,57,60] constrain the weight of the findings. Methodological heterogeneity and potential confounding factors present notable challenges [87,88]. Adopting standardised research protocols and transparent reporting practices is imperative to address these issues. Furthermore, research in this area has predominantly focused on specific racial or ethnic groups, potentially limiting its generalisation to other populations [88].

Future research should prioritise larger cohorts with elite athletes to reinforce its statistical power. While genetic association studies related to disease have an average of 8500 cases, performance-related studies have an average of 382 participants. To address this problem, future research should prioritise larger cohorts with elite athletes to reinforce the statistical power [89]. Future research should encompass genome-wide association (GWA) studies and replication studies, confirming the significance of SNPs through meta-analyses [28,90] and focusing on the polygenic nature of combat attributes. The identification of the genetic variants that increase injury susceptibility may permit the future tailoring of an athlete’s training to a suitable level and enhance their recovery from injury, enabling young athletes to have a more prosperous and potentially longer career [91]. However, we are still far from the full identification of all injury-influencing genetic variants, and the evidence base identifying the variants and their modality of effect (protective, causal, etc.) needs to be considerably stronger than it is currently.

In conclusion, this systematic review explored the role of genetics in combat sports, investigating the intricate relationship between genetic factors and diverse performance determinants. The systematic review revealed key genetic variations associated with physiological, psychological, and injury-related aspects. Despite inherent limitations, such as small sample sizes and methodological heterogeneity, the findings invite hope and speculation regarding the possibilities of tailored training, injury prevention, and talent identification in combat sports. Integrating various omics approaches, such as genomics, epigenomics, transcriptomics, proteomics, and metabolomics, could provide a holistic understanding of the biological mechanisms underpinning individual athletes’ training, nutrition, and recovery strategies. However, as mentioned above, most of the studies to date have been small and often associational in nature. To be able to fully realise the potential of integrating this information into athlete training programs, there need to be larger-cohort, replicative studies and mechanistic investigations with various efficacy models, simultaneously balanced with a wider understanding of the ethical implications from a range of perspectives (e.g., wider sporting and non-sporting communities, individual athletes, training organisations, and both local and global sports governance bodies).

## Data Availability

All data analysed in this study are included in the tables.
